# Characteristics and sexual health service use of MSM engaging in chemsex: results from a large online survey in England

**DOI:** 10.1136/sextrans-2019-054345

**Published:** 2020-03-05

**Authors:** Paula Bianca Blomquist, Hamish Mohammed, Amy Mikhail, Peter Weatherburn, David Reid, Sonali Wayal, Gwenda Hughes, Catherine H Mercer

**Affiliations:** 1 UK Field Epidemiology Training Programme, Global Public Health Division, Public Health England, London, United Kingdom; 2 Field Service North West, National Infection Service, Public Health England, Liverpool, United Kingdom; 3 National Institute for Health Research Health Protection Research Unit (NIHR HPRU) in Blood Borne and Sexually Transmitted Infections at University College London in partnership with Public Health England (PHE), in collaboration with London School of Hygiene & Tropical Medicine, London, United Kingdom; 4 Blood Safety, Hepatitis, Sexually Transmitted Infections (STI) and HIV Division, Public Health England, London, United Kingdom; 5 Centre for Population Research in Sexual Health and HIV, Institute for Global Health, University College London, London, United Kingdom; 6 Sigma Research, Department of Public Health, Environments & Society, London School of Hygiene and Tropical Medicine, London, United Kingdom

**Keywords:** sexual health, gay men, injecting drug use, HIV, sexual behaviour

## Abstract

**Background:**

Chemsex, the use of select psychoactive drugs to enhance sexual experience, typically among men who have sex with men (MSM), is associated with sexual behaviours with higher STI risk. Understanding patterns of chemsex among MSM as well as the characteristics and sexual health service engagement of chemsex participants is important for developing interventions.

**Methods:**

Between 5/2016 to 5/2017, 3933 MSM completed an online survey, recruited in sexual health clinics (SHCs) in England (n=421) and via four social networking/dating apps (n=3512). We described patterns of chemsex in the past year and used multivariable logistic regression to investigate differences in demographics and sexual behaviours by chemsex history. We described history of SHC attendance and STI test in the past year among app-recruited chemsex participants.

**Results:**

Chemsex in the past year was reported by 10% of respondents; 19% of SHC-recruited and 9% of app-recruited. Among chemsex participants, 74% had used ≥2 chemsex drugs. In the multivariable model, MSM engaging in chemsex had a raised odds of being HIV-positive (adjusted OR (aOR): 3.6; 95% CI 2.1 to 6.1), aged 30–44 (aOR 1.5 vs <30 years; 95% CI 1.0 to 2.1), being born outside the UK and having engaged in higher risk sexual behaviours in the past 3 months. Chemsex participants also had higher odds of condomless anal sex with partners of different or unknown HIV status, but only among HIV-negative/untested. In the past year, 66% of app-recruited chemsex participants had attended a SHC and 81% had had an STI test.

**Conclusion:**

One in 10 MSM recruited through community and clinical settings across England had engaged in chemsex in the past year. Those that did appear to be at greater STI risk but engaged more actively with sexual health services. This highlights the need and opportunity for chemsex-related services in SHCs and robust referral pathways to drug treatment services.

## Introduction

Chemsex refers to the use of specific recreational drugs before and/or during sexual activity to enhance the sexual experience of gay, bisexual and other men who have sex with men (MSM).^1^ The drugs, typically mephedrone, gamma-hydroxybutyrate/gamma-butyrolactone (GHB/GBL), crystal methamphetamine and to a lesser extent ketamine,^1 2^ encourage disinhibition and enhance libido and stamina.^3 4^

Chemsex is a recognised public health concern. As well as risks of drug addiction and harms arising from injecting drug use, there is increased potential for transmission of sexually transmitted infections (STIs) and HIV as well as other bloodborne viruses such as hepatitis C.^4^ This is due to the enhanced drive and ability to have multiple sex partners over a short time, often without condoms and with additional higher risk sexual practices (e.g., fisting).^1 2 4^ These health risks are likely facilitated by the use of social networking/dating apps (simplified here to ‘apps’) to find sex partners.^2^

However, chemsex does not always mean harm to an individual’s sexual health. Controlled drug use and risk management are possible.^5^ Risk of HIV/STI transmission for instance can be mitigated by condom use during chemsex sessions and timely access to STI screens or sexual health advice. Understanding the prevalence of chemsex and the characteristics, sexual behaviours and healthcare access of those who engage in it is important for understanding the health risks associated with chemsex and for designing interventions to address them.

Prior to this work, research into chemsex prevalence among MSM in England, and their characteristics, had been undertaken in mostly clinic-based studies or studies with few sites.^1 4 6^ One study looked into sexual health service engagement among community-representative chemsex participants in London, England, and found that 70% of HIV-negative respondents had attended a sexual health clinic (SHC) in the last year.^6^ A survey across the UK (excluding England) and Ireland found that 64% of chemsex-participating MSM had had an HIV test in the past year.^7^ Additionally, a survey distributed to SHCs in the UK found that half of healthcare workers had a chemsex consultation at least monthly.^8^

The aim of this study was to better understand the degree to which MSM in England engage in chemsex and the associated demographic and behavioural factors. We also determined the proportion of MSM engaging in chemsex who attended SHCs or had an STI test.

## Methods

### Study design

Data were collected for the Reducing Inequalities and Improving Sexual Health study, undertaken 2016–2017 for the National Institute for Health Research Health Protection Research Unit for Blood Borne and Sexually Transmitted Infections. This cross-sectional study administered an online survey to MSM in clinical settings and via apps. Comprehensive methodology details have previously been published^9^ and are summarised below.

### Study setting

The clinical setting comprised of 16 SHCs across England with particularly high proportions of MSM attendees, in London, Birmingham, Manchester and Leeds. Between May and September 2016, staff handed out paper invitations to SHC attendees, on which the survey weblink was printed. Respondents could complete the survey in the clinic using the study tablet or at any time on their own internet-enabled device (eg, smart phone). The questionnaire collected information on demographics, healthcare-seeking behaviour, sexual behaviour and drug use.

Between March and May 2017, the same survey was promoted via three apps (Scruff, Gaydar, Grindr) which displayed a pop-up message with a survey link. The link was also posted on the home page for Sigma Research, one of the research centres affiliated with this study (http://sigmaresearch.org.uk/). No incentives were offered for either recruitment method.

### Eligibility criteria

Screening questions at the beginning of the questionnaire filtered to eligible respondents: men aged at least 16, who reported sex with a man in the last 12 months, and who consented to participate in the survey. In the app version of the survey, an additional screening question asked if respondents had previously completed the survey in clinic, and these men were excluded to prevent duplication.

### Analysis

#### Description of study population

We first described the study population in terms of overall numbers and distribution of demographic characteristics, stratified by recruitment method.

#### History of chemsex in 12 months prior to survey

We described the proportion of all respondents who reported using a chemsex drug in the past 12 months, here defined as crystal methamphetamine, mephedrone, GHB/GBL or ketamine. We then described the proportions of respondents who had chemsex in the past 12 months, which we defined as having sex after taking any of the above drugs. For simplicity, we do not always explicitly refer to the 12 months in this paper. We stratified results by HIV status.

#### Comparison of demographic factors and sexual behaviour by chemsex participation in past 12 months

To investigate factors associated with chemsex, we presented the distribution of demographic factors and sexual behaviours among MSM who did and did not report chemsex in the past 12 months. Demographic factors were captured by the following categorical variables: age group, ethnic group, continent of birth, highest educational qualification, and HIV status. Ethnicity groupings were based on broad categories used by the UK’s Office for National Statistics (who use White, Black, Asian, Mixed, and Other).^10^ Sexual behaviour was captured by the following variables on sex with men in the past 3 months: binary variables for if they had a new male partner or had condomless anal sex (CAS) and categorical variables on number of partners and HIV serostatus of partners among those reporting CAS (all partners’ statuses known and all seroconcordant, all known and some serodiscordant, and at least one serostatus unknown).

We conducted a univariable logistic regression analysis to compare these characteristics by chemsex participation, reporting crude odds ratios (ORs) and global p values. To address the interplay between these factors, we then constructed a multivariable logistic regression model based on variables where p values from the univariable analysis were <0.05. We used a forward-building method introducing variables in order of highest to lowest measure of effect and maintained variables if their addition to the model resulted in an improvement of fit with a likelihood ratio test p<0.05. We checked for evidence of substantial confounding, demonstrated by change to an OR by >10% when adding a new variable; if the variable no longer had a significant association (at p<0.05) with the outcome of chemsex it was dropped. Once the variables in the multivariable model were decided, we checked for two-way effect modification due to HIV status. Only plausible interactions were checked, and interaction terms were maintained in the model if significant at p<0.05.

Finally, to check for clustering among individuals recruited in the same site, we added a random intercept (a categorical variable distinguishing between apps and specific SHCs) and conducted a likelihood ratio test to determine if this improved the model (if p<0.05).

#### History of SHC attendance by chemsex use among respondents recruited via apps

We investigated the proportion of respondents who had attended a SHC or had an STI test in the 12 months prior to completing the survey, by history of chemsex. This analysis was restricted to men recruited via apps, as including SHC-recruited men would have overestimated SHC attendance.

Data were analysed using Stata (V.14.2). All percentages are presented with 95% CIs and are based on non-missing responses. Where appropriate, we report missingness of data.

## Results

### Description of study population

The survey was completed by 3933 eligible MSM, of which 421 were recruited in SHCs and 3512 via apps: 54% Grindr, 44% Gaydar, 2% Scruff and <1% the Sigma webpage (online supplementary material figure 1).

10.1136/sextrans-2019-054345.supp1 Supplementary data

The median respondent age was 43 years (IQR 31–53 years), 90% identified as white ethnicity, 19% were non-UK born and 52% were university educated. Clinic survey respondents were younger and were more likely to be university educated, born outside the UK, and of non-white ethnicity (p<0.001; detail in online supplementary material table 1). Overall, 14% (n=557) of respondents reported being diagnosed with HIV, although this was higher in the SHC-recruited group (23% vs 13%, p<0.001). In our sample, HIV-positive persons were slightly older (median age 47 vs 43) and more likely to be white than HIV-negative/untested (94% vs 90%, p=0.002).

### History of chemsex in 12 months prior to survey

**Figure 1:**
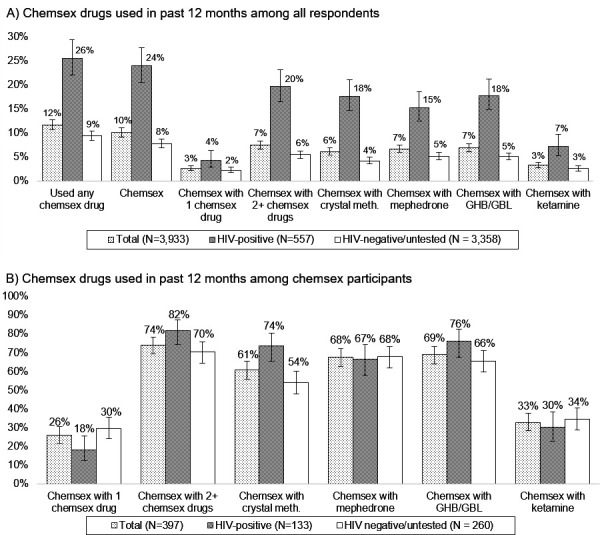
Chemsex drugs used in the 12 months prior to completing RiiSH MSM survey (2016–2017) among (A) all respondents and (B) respondents reporting chemsex in the past 12 months, in total and by HIV status. Non-missing percentages to 0 decimal places and CIs presented. GBL, gamma-butyrolactone; GHB, gamma-hydroxybutyrate; MSM, men who have sex with men.

Chemsex status was documented for 3922 respondents (99.7% completeness). As figure 1 shows, 11.7% reported using at least one chemsex drug in the past 12 months. Among them (n=460), 87.6% used these drugs for chemsex. This equated to 10.1% of all respondents.

Chemsex varied by recruitment method: 19.2% of SHC-recruited respondents reported chemsex in the past 12 months compared with 9.0% of app-recruited individuals. Chemsex participation was also distinctly higher among HIV-positive (23.9%) than HIV-negative/unknown MSM (7.8%; figure 1A).

Among the 397 MSM reporting chemsex (figure 1B), 74.1% reported using two or more chemsex drugs, whereas 25.9% used only one. This was slightly higher in HIV-positive respondents (n=133): 82.0% and 18.1% respectively. Use of GHB/GBL was most commonly reported (68.9% of chemsex participants) while ketamine was least frequently reported (32.8%). Specific drug preferences differed little by HIV status, except that crystal methamphetamine use was higher in HIV-positive MSM.

### Comparison of demographic factors and sexual behaviour by chemsex participation in past 12 months

#### Univariable analysis

Our univariable analysis found associations between chemsex and all investigated variables (table 1). Compared with MSM who did not report chemsex, a higher proportion of chemsex participants were aged 30–44 (40.3% vs 30.9%), university educated (59.5% vs 51.3%), and HIV-positive (33.8% vs 12.0%). While approximately 90% of both groups identified as white ethnicity, more chemsex-reporting MSM identified specifically as ‘white other’ (17.6% vs 9.0%) and were born outside the UK.

**Table 1:** Demographic characteristics and sexual behaviour in 3 months prior to completing RiiSH MSM survey (2016–2017), by chemsex participation in the past 12 months

	Non-missing percentage			Univariable logistic regression		
	No chemsex last 12 months (n=3525) (%)	Chemsex in last 12 months (n=397) (%)	Total (n=3933) (%)	OR	CI	Global p value
Recruitment method						
App survey	90.4	79.8	89.3	–	–	
Clinic survey	9.6	20.2	10.7	2.39	1.82 to 3.13	<0.001
Age group						
16–29	21.1	17.6	20.7	–	–	
30–44	30.9	40.3	31.9	1.57	1.16 to 2.10	<0.001
45–59	36.0	36.0	36.0	1.20	0.88 to 1.62	
60+	12.0	6.0	11.4	1.20	0.37 to 0.98	
Ethnic group*						
White British/Irish	81.3	71.8	80.2	–	–	
White other	9.0	17.6	10.0	2.20	1.65 to 2.94	0.049
Black	2.1	2.5	2.2	1.36	0.69 to 2.66	
Asian	4.0	4.3	4.1	1.23	0.73 to 2.07	
Mixed ethnicity	2.5	3.3	2.6	1.52	0.83 to 2.26	
Other	1.1	0.5	1.0	0.55	0.13 to 2.27	
Continent of birth*						
UK	83.0	72.3	81.9		–	
Africa	2.0	3.6	2.2	2.00	1.11 to 3.60	<0.001
Asia	3.4	3.1	3.3	1.04	0.56 to 1.91	
Australasia	1.0	2.8	1.2	3.19	1.60 to 6.36	
Mainland Europe	7.9	14.5	8.6	2.11	1.54 to 2.87	
North America	1.2	1.5	1.3	1.42	0.59 to 3.36	
South America	1.4	2.3	1.5	1.87	0.90 to 3.84	
Highest qualification received*						
Below degree	48.7	40.5	47.8	–	–	
University degree or higher	51.3	59.5	52.2	1.40	1.13 to 1.73	0.002
HIV status*						
No known HIV diagnosis	88.0	66.2	85.8	–	–	
HIV-positive	12.0	33.8	14.2	3.74	2.96 to 4.71	<0.001
No. male partners in past 3 months†						
None	11.2	4.4	10.6	–	–	
1–5	64.9	45.3	62.9	1.78	1.05 to 3.02	<0.001
6–10	13.3	20.9	14.1	4.00	2.29 to 6.98	
11+	10.5	29.4	12.4	7.16	4.15 to 2.37	
New male sexual partner in past 3 months†						
No	33.0	17.1	31.4	–	–	
Yes	67.0	82.9	68.6	2.38	1.78 to 3.19	<0.001
CAS with man in past 3 months*						
No	51.2	24.1	48.4	–	–	
Yes	48.8	75.9	51.6	3.30	2.59 to 4.2	<0.001
HIV status of CAS partners(s) in past 3 months among those reporting CAS (no chemsex n=1676, chemsex n=296)‡						
HIV status known: seroconcordant partner(s) only	60.0	48.1	58.1	–	–	
HIV status known: at least one serodiscordant partner	19.7	28.3	21.1	1.79	1.30 to 2.47	0.001
Unknown HIV serostatus in at least one partner	20.3	23.6	20.8	1.46	1.04 to 2.04	
Total	100.0	100.0	100.0			

Non-missing percentages presented.

*Variables have <2.5% missing data with no difference by chemsex participation.

†Variables have 9%–15% missing data with no difference by chemsex participation.

‡Variable has 17.9% missing data among persons reporting CAS: 12% and 18% of those who did and did not report chemsex.

CAS, condomless anal sex; MSM, men who have sex with men; RiiSH, Reducing Inequalities and Improving Sexual Health.

In terms of sexual behaviour, a higher proportion of chemsex-reporting MSM reported each of the following in the past 3 months: >10 male sexual partners (29.4% vs 10.5%), a new male partner (82.9% vs 67.0%), and CAS (75.9% vs 48.8%). Among those who reported CAS, a higher proportion of chemsex participants (n=296) reported CAS with at least one partner with a different (28.3% vs 19.7%) or unknown HIV status (23.6% vs 20.3%).

#### Multivariable logistic regression

**Figure 2:**
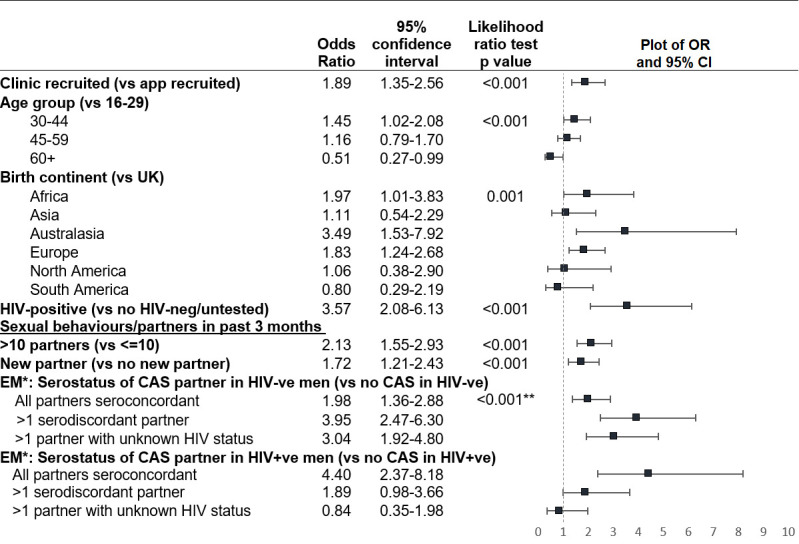
Multivariable logistic regression results: demographic factors and sexual behaviours associated with chemsex in the 12 months prior to completing RiiSH MSM survey (2016–2017). Adjusted ORs and 95% CIs presented to two decimal places. *Effect modification: calculated ORs shown within HIV-positive and HIV-negative/untested subgroups; **P value for interaction shown. CAS, condomless anal sex; MSM, men who have sex with men; RiiSH, Reducing Inequalities and Improving Sexual Health.

In the multivariable model, the odds of chemsex were raised in those aged 30–44, born in mainland Europe, Australasia or Africa, and HIV-positive (figure 2). Ethnic group and university education were no longer associated with chemsex, as these were confounded by birth continent (detail in online supplementary material table 2).

MSM engaging in chemsex had a 2.1 times higher adjusted odds of >10 sexual partners in the past 3 months compared with MSM who did not report chemsex (95% CI 1.6 to 2.9), and a 1.7 times higher adjusted odds of a new male sexual partner in the last 3 months (95% CI 1.2 to 2.4).

Due to collinearity, the final multivariable model used a composite variable combining history of CAS and HIV status of CAS partner(s) in the past 3 months. MSM engaging in chemsex had a higher odds of reporting CAS, particularly with a seroconcordant partner, however the effect was modified by HIV status. When comparing HIV-negative/untested men who did and did not engage in chemsex, those who reported chemsex had a 2.0 times higher odds of engaging in CAS only with other known HIV-negative partners and even higher odds of CAS with a HIV-positive partner (adjusted OR (aOR): 4.0 95% CI: 2.5 to 6.3) or partner of unknown HIV status (aOR: 3.0, 95% CI: 1.9 to 4.8). Among HIV-positive MSM, those engaging in chemsex had a 4.4 times higher odds of engaging in CAS with only HIV-positive partners (95% CI 2.4 to 8.2), but there was no evidence of a higher odds of sex with partner(s) of unknown status or who were HIV-negative. The corresponding percentages for these interacting variables are in online supplementary material table 3.

We did not add a random effect for specific recruitment site, as the mixed effects model did not improve the fit (p=1.00), indicating homogeneity of outcome between clinics.

### Sexual health service use in the past 12 months among app-recruited respondents

**Figure 3:**
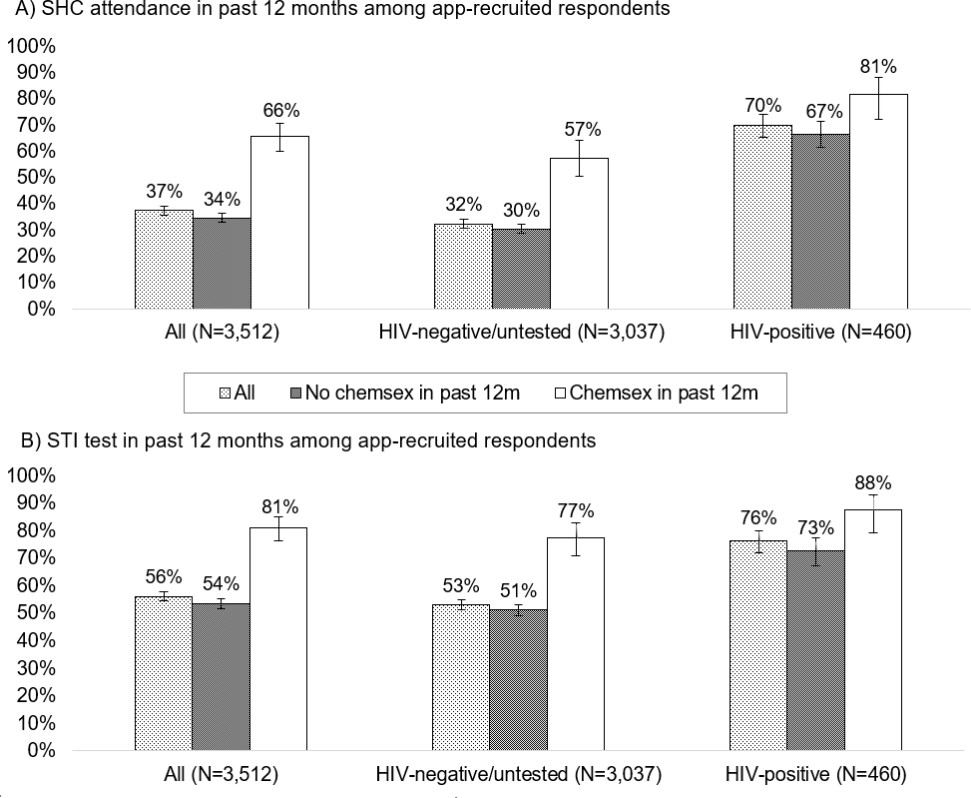
Proportions of MSM reporting (A) a SHC attendance and (B) an STI test in the 12 months prior to completing the RiiSH MSM survey (2016–2017), by HIV status and history of chemsex of the past 12 months. Non-missing percentages to 0 decimal places and 95% CIs presented. MSM, men who have sex with men; RiiSH, Reducing Inequalities and Improving Sexual Health; SHC, sexual health clinic.

SHC attendance and STI testing history were reported by 3232 (92.1%) and 3109 (88.5%) of app-recruited respondents, respectively. In the past 12 months, 37.4% had attended a SHC and 56.1% had had an STI test (figure 3). These percentages were higher among chemsex participants (65.5% attended a SHC and 81.0% had an STI test) and even higher among HIV-positive chemsex participants (81.4% and 87.6%, respectively).

## Discussion

In this large multicentre study of MSM recruited from apps and SHCs across England, 1 in 10 men reported engaging in chemsex in the past 12 months. Chemsex was more common among specific subgroups: one in four HIV-positive MSM and one in five MSM recruited via SHCs.

We confirmed that use of crystal meth, mephedrone, GHB/GBL, and ketamine is mostly sexualised among MSM. Furthermore, three of four chemsex participants used multiple chemsex drugs, although it is unknown if they were used in the same session.

MSM engaging in chemsex were more likely to be clustered in the 30–44 age group, be born in Australasia, mainland Europe or Africa, have multiple or new sexual partners, and engage in CAS. Chemsex participants were more likely to report partners with a different or unknown HIV status, but only among the HIV-negative/untested. Chemsex was not associated with ethnicity or education, as these relationships were confounded by continent of birth: MSM originating from non-UK countries were more educated.

Finally, most MSM engaging in chemsex in the past 12 months had attended a SHC or had an STI test in the corresponding period. The higher healthcare engagement among HIV-positive MSM likely reflects HIV-related care.

This was a large study and the only one we are aware of to investigate chemsex among respondents recruited from both apps and SHCs in cities across England. It is also one of the first to ask community-recruited chemsex-reporting MSM in England about SHC use.

However, as this is a cross-sectional survey, we do not know if the SHC attendance took place before or after chemsex. We did not collect information on chemsex-related health problems, so cannot estimate the need for sexual health or drugs services in this population. Although pre-exposure prophylaxis (PrEP) for HIV was becoming available during our study,^11^ we did not ask about it, and this may have been a relevant factor influencing sexual behaviour and partner choice.

Our study findings are not necessarily generalisable to MSM across England. App users may be more engaged with technology, more sexually active and more health-aware than the general MSM population. However, there is evidence that MSM recruited online are more representative geographically of the general MSM population than those recruited in SHCs.^12^ Finally, we may have under-represented individuals born outside the UK, as the survey was only in English.

Our findings of 9% app-recruited MSM reporting chemsex are similar to prevalence estimates in some community-based studies: 9% among Brighton MSM^13^ and 8% among app-recruited MSM in the UK (excluding England) and Ireland,^7^ although lower than the 21% among HIV-negative MSM in London.^6^ Additionally, our 19% chemsex estimate among SHC-recruited MSM falls within the range seen in other SHC-focused studies in England.^1 4^ However, the sampling methods, chemsex definitions, and look-back periods differ. Our study is also more representative of SHCs across England. Importantly, while these are two separate recruitment methods, these are not mutually exclusive populations: for example, some SHC-recruited persons will also be app users and vice versa.

Other works have also noted that chemsex-participating MSM in England are mostly in their 30s and 40s, white, highly educated and report greater sexual risk behaviours.^4 6^ However, this study has also explored how HIV status is associated with these behaviours and how ethnicity and education are confounded by birth continent. These analyses were enabled by our larger sample size.

Finally, the high engagement with SHCs among study respondents, particularly those reporting chemsex, has been seen elsewhere^4 6 7^ and is consistent with research demonstrating that chemsex-participating MSM are comfortable accessing SHCs^14^ and chemsex-related consultations in SHCs are common.^8^ However, we cannot know how much chemsex was addressed in the SHC visits reported by our study respondents.

Chemsex was not widespread among study respondents, but the associated sexual behaviours demonstrate the need for chemsex participants to engage with sexual health services. Fortunately, we did find SHC visits and STI testing to be higher in this group, indicating risk management through either proactive check-ups and/or responses to symptoms/known exposures. This also corroborates with our finding that chemsex was higher among those recruited in SHCs compared with apps.

The generally high SHC use presents a viable opportunity to provide chemsex-related sexual health services, particularly in venues providing HIV care. Considering findings from other research that only a third of SHCs have chemsex care-pathways in place,^8^ there have likely been missed opportunities to address wider health needs such as mental health and other drug-related harms.

Healthcare professionals should harness visits to SHCs to identify and support chemsex-related needs, through better integration or connectivity between drug/alcohol and sexual health services. While integration may be unrealistic in smaller clinics or challenging due to differences in commissioning,^3^ basic training of SHC staff in drugs and alcohol and the setup of referral systems and effective signposting is feasible and useful. These synergies should not be limited to SHCs: the higher proportion of individuals that had an STI screen than had visited a SHC implies that alternative services are being used. Information or resources on chemsex should be available via these avenues, for example, links on self-sampling/self-testing websites.

Chemsex services should be culturally competent^5^ and address patient needs and demographics, for instance that chemsex participants are more likely to originate from outside the UK. SHCs in England have long been praised for convenient, anonymous, and non-judgmental care. Investments to adapt to changing needs of attendees are needed to ensure they stay this way.

**Key messages**

- One in 10 men who have sex with men recruited through community and clinical settings across England had engaged in chemsex in the past year.
- Chemsex participants were more likely to be HIV-positive, aged 30–44, born outside the UK and report higher risk sexual behaviours.
- Among app-recruited respondents reporting chemsex, 66% had attended a sexual health clinic and 81% had had an STI test in the past year.
- Results highlight the need and opportunity for chemsex-related services in sexual health clinics and referral pathways to drug treatment services.
