# Supplementary material for: Characteristics and sexual health service use of MSM engaging in chemsex: results from a large online survey in England
Source: Sex Transm Infect. 2020 Mar 5;96(8):590–5. doi: 10.1136/sextrans-2019-054345 (PMC7677472; doi:10.1136/sextrans-2019-054345)
Supplement: Supplementary data [file sextrans-2019-054345supp001.pdf]

Characteristics and sexual health service use of MSM engaging in chemsex: results from a large online survey in England - Supplementary material

Figure 1: Flow chart of survey recruitment from sexual health clinics (left) and apps (right)

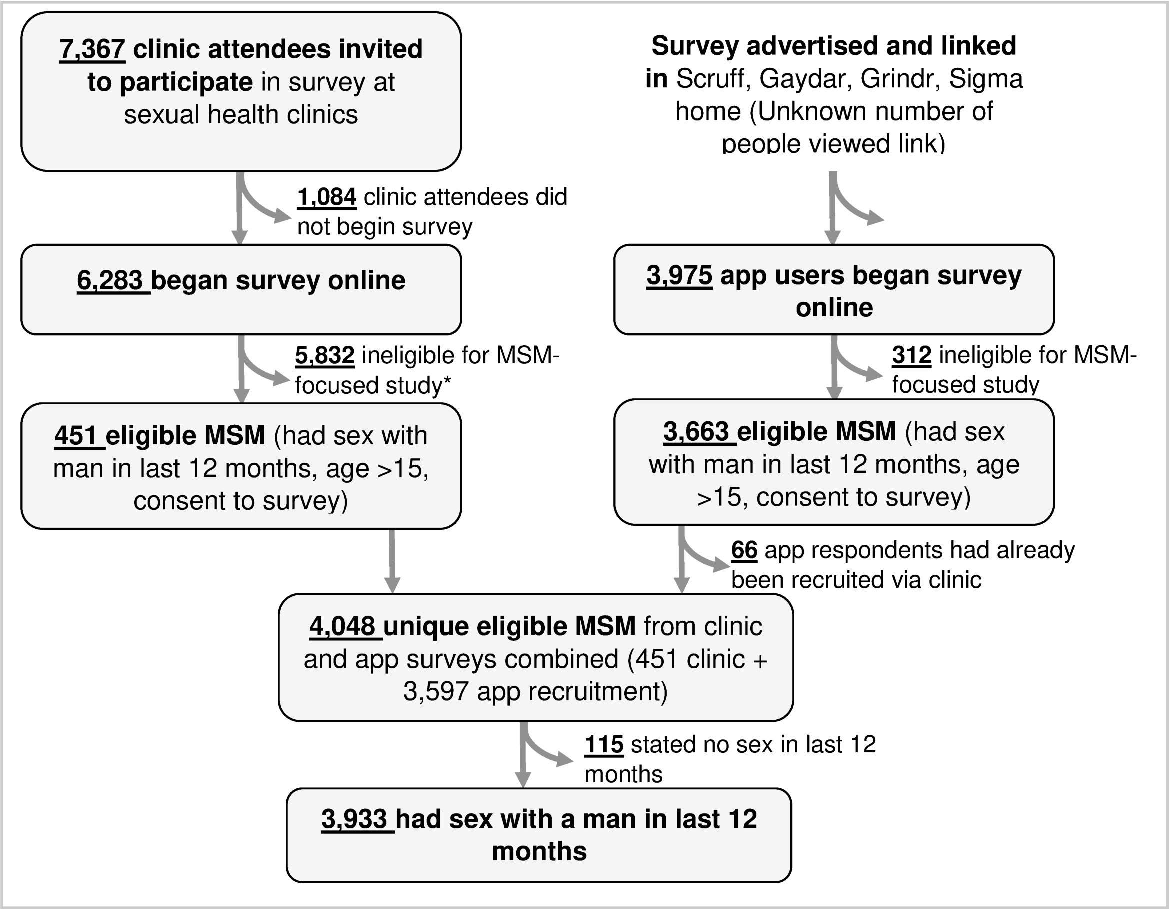

**Note that the low proportion of eligible SHC-recruited respondents reflects that women and heterosexual men were also invited to the overall RiiSH study, however they were routed to a different questionnaire for heterosexual respondents which is not relevant to this paper.*

Table 1: Comparison of demographic characteristics among respondents to the RiiSH survey recruited from sexual health clinics and from social networking/dating apps in England (2016-2017). Non-missing percentages presented.

	Number			Non-missing percentage			
	Clinic recruited	App recruited	Total	Clinic recruited	App recruited	Total	**Chi2 p value**
**Age group**							
16-29	181	638	819	**43.0%**	**18.2%**	**20.8%**	<0.001
30-44	163	1,090	1,253	**38.7%**	**31.0%**	**31.9%**	
45-59	66	1,349	1,415	**15.7%**	**38.4%**	**36.0%**	
60+	11	435	446	**2.6%**	**12.4%**	**11.3%**	
**Ethnicity***							
White British/Irish	267	2,856	3,123	**64.0%**	**82.2%**	**80.2%**	<0.001
White other	55	333	388	**13.2%**	**9.6%**	**10.0%**	
Black	30	54	84	**7.2%**	**1.6%**	**2.2%**	
Asian	31	127	158	**7.4%**	**3.7%**	**4.1%**	
Mixed ethnicity	23	77	100	**5.5%**	**2.2%**	**2.6%**	
Other	11	28	39	**2.6%**	**0.8%**	**1.0%**	
**Country of birth***							
Not UK	134	583	717	**31.9%**	**16.9%**	**18.5%**	<0.001
UK	286	2,876	3,162	**68.1%**	**83.1%**	**81.5%**	
**Highest qualification received***							
Below degree	153	1,693	1,846	**36.4%**	**49.2%**	**47.8%**	<0.001
Uni degree or higher	267	1,745	2,012	**63.6%**	**50.8%**	**52.2%**	
**HIV status***							
HIV-negative/untested	321	3,037	3,358	**76.8%**	**86.8%**	**85.8%**	<0.001
HIV-positive	97	460	557	**23.2%**	**13.2%**	**14.2%**	
**Sex app**							
Grindr	-	1,894	1,894	-	**53.9%**	**48.2%**	-
Gaydar	-	1,527	1,527	-	**43.5%**	**38.8%**	
Scruff	-	67	67	-	**1.9%**	**1.7%**	
Sigma website	-	20	20	-	**0.6%**	**0.5%**	
**Total**	421	3,512	3,993	**100.0%**	**100.0%**	**100.0%**	

**Variables have <2.5% missing data with no difference by recruitment method*

Table 2: Univariable logistic regression analysis results on association between highest qualification received and chemsex in the 12 months prior to completing RiiSH MSM survey (2016-2017), stratified by continent of birth (simplified to UK vs non-UK; Non-missing percentages presented)

	No chemsex in past 12 months		Chemsex in past 12 months		Univariable logistic regression		
	n	%	n	%	Odds ratio	95% confidence interval	Global p value
**All**							
Not university educated	1683	48.7%	159	40.5%	1.40	1.13-1.73	0.002
University educated	1771	51.3%	234	59.5%			
**Born outside UK**							
Not university educated	164	28.3%	26	24.1%	1.24	0.77-2.00	0.371
University educated	416	71.7%	82	75.9%			
**Born in UK**							
Not university educated	1500	53.1%	132	47.0%			
University educated	1326	46.9%	149	53.0%	1.27	0.99-1.63	0.510

Table 3. History of condomless anal sex (CAS) and HIV status of partner(s) in 3 months prior to completing RiiSH MSM survey (2016-2017), by HIV status and chemsex participation in the past 12 months. Non-missing percentages presented

	No chemsex in past 12 months		Chemsex in past 12 months	
	n	%	n	%
**HIV-positive***				
No CAS	164	43.7%	24	20.5%
All partners seroconcordant	68	18.1%	52	44.4%
>1 serodiscordant partner	87	23.2%	29	24.8%
>1 partner with unknown HIV status	56	14.9%	12	10.3%
**HIV-negative/unknown***				
No CAS	1586	58.1%	70	30.0%
All partners seroconcordant	747	27.4%	72	30.9%
>1 serodiscordant partner	180	6.6%	42	18.0%
>1 partner with unknown HIV status	218	8.0%	49	21.0%

**Missingness similar across all groups: HIV-positive reporting no chemsex: 11.3%; HIV-positive reporting chemsex:12.0%, HIV-negative/unknown reporting chemsex: 10.4%; HIV-negative/untested reporting no chemsex: 11.6%. No identified demographic differences between persons who did and did not respond.*
